# Bayesian Inference for Functional Dynamics Exploring in fMRI Data

**DOI:** 10.1155/2016/3279050

**Published:** 2016-03-01

**Authors:** Xuan Guo, Bing Liu, Le Chen, Guantao Chen, Yi Pan, Jing Zhang

**Affiliations:** ^1^Department of Computer Science, Georgia State University, Atlanta, GA 30303, USA; ^2^Department of Mathematics and Statistics, Georgia State University, Atlanta, GA 30303, USA; ^3^Department of Biology, Georgia State University, Atlanta, GA 30303, USA

## Abstract

This paper aims to review state-of-the-art Bayesian-inference-based methods applied to functional magnetic resonance imaging (fMRI) data. Particularly, we focus on one specific long-standing challenge in the computational modeling of fMRI datasets: how to effectively explore typical functional interactions from fMRI time series and the corresponding boundaries of temporal segments. Bayesian inference is a method of statistical inference which has been shown to be a powerful tool to encode dependence relationships among the variables with uncertainty. Here we provide an introduction to a group of Bayesian-inference-based methods for fMRI data analysis, which were designed to detect magnitude or functional connectivity change points and to infer their functional interaction patterns based on corresponding temporal boundaries. We also provide a comparison of three popular Bayesian models, that is, Bayesian Magnitude Change Point Model (BMCPM), Bayesian Connectivity Change Point Model (BCCPM), and Dynamic Bayesian Variable Partition Model (DBVPM), and give a summary of their applications. We envision that more delicate Bayesian inference models will be emerging and play increasingly important roles in modeling brain functions in the years to come.

## 1. Introduction

An intriguing quest regarding the brain science is the following: what are the origin and the principles behind the functional architectures, which define who we are and what we are in a great extent? Compared to other methods, functional Magnetic Resonance Imaging (fMRI) has been well recognized as the most popular method that is able to explore the functional activities of the whole brain when participants are in resting states (have a rest) or performing a carefully designed task, due to its in vivo and noninvasive nature. After decades of active research, there have been numerous evidences [1–5] that the brain function is realized and emerges from the interaction of multiple concurrent neural processes or brain networks, each of which is spatially distributed across different neuroanatomical areas and exhibits statistically interdependent activity patterns. Recently, researches on brain functional connectivity or interactions based on fMRI BOLD (fMRI blood-oxygen-level dependent) signals have received substantial interests. In previous studies, the functional connectivity was assumed to be temporal stationery [6–9]. However, the emerging evidence from neuroscience research indicates that different cortical regions are acting as adaptive processors, which involve moment-by-moment functional switching. Many studies implied that the function of different cortical regions is subject to top-down influences of brain cognitive process [10]. Task-based studies have also revealed that cognitive processes, like attention and learning, might significantly change in functional connectivity when performing tasks across the duration of a scan [11–13]. In addition, recent works suggest that fine-grained brain subnetworks, operating as communication hubs in graph theoretical terms, are equally involved in task performance [14, 15]. Besides functional connectivity, functional network connectivity (FNC), which studies the interactions in network level, requires estimating the clusters of brain regions having similar functions and has been applied to many diseases to examine brain network differences between healthy and diseased brains [16–18]. For instance, by using a maximal lagged correlation approach, changes in FNC were found in schizophrenia, a brain disorder that is known as disrupted cognitive functions [19, 20].

In current stage, it is still challenging to infer the temporal dynamics of functional connectivity, which can be summarized as two points: identification of change points and estimation of dynamic interaction patterns. There are accumulating studies analyzing the brain functional dynamics, connectivity dynamics, and the state changes. Lindquist et al. proposed a statistical method, named as Hierarchical Exponentially Weighted Moving Average (HEWMA), on fMRI data to detect the state change of BOLD signals in response to stimulus [21]. Independent component analysis (ICA) method, including dynamic spatial ICA, was developed by Sakoğlu et al. to investigate the connectivity dynamics [20]. Also, sliding-time-window-based approaches were designed to capture the dynamics of brain functional interactions across different time windows [22–27]. For example, Allen and colleagues described an approach to assess whole-brain FC dynamics based on spatial ICA, sliding-time-window correlation analysis, and *k*-means clustering of windowed correlation matrices [27], and it revealed unanticipated FC states which were strongly different from stationary connectivity patterns. Li and his colleagues derived functional connectomes (FCs) to characterize brain conditions from resting-state fMRI data and then FCs were divided into quasi-stable segments temporally via a sliding-time-window approach [22]. In [23], a novel framework was designed by the combination of sliding-window approach and multiview spectral clustering to extract temporally dynamic functional connectome patterns for resting-state networks, and the four detected clusters are believed to play critical roles in functional brain dynamics during resting states. In [24], a novel algorithmic framework based on hidden Markov models was presented to cluster and label the brain's functional states, represented by a large-scale functional connectivity matrix and derived via an overlapping sliding-time-window approach. The framework achieved decent classification performance on the data including 25 ADHD (attention-deficit/hyperactivity disorder) patients and 49 normal controls. Lv and his colleagues [25] also adopted the sliding-window-based method and employed a dynamic programming strategy to infer functional information transition routines on structural networks and identified the hub routers that participate in these routines most frequently.

Inspired by the multivariate graphical models based on Bayesian networks, which has been shown to be robust and reliable in estimating functional interactions and less sensitive to noise in the fMRI signals [28–30], recently, several Bayesian-inference-based methods were proposed to infer global functional interactions within brain networks and their temporal transition boundaries [31–36]. By evaluating and estimating both simulated and real data, Bayesian-inference-based methods are proved to be more powerful approaches for analysis fMRI data comparing to the methods mentioned above.

In this paper, we are aiming to pinpoint the function dynamic problems, including detecting magnitude change points, functional connectivity change points, and functional interaction patters, which have been addressed using Bayesian inference. The organization of this paper is as follows. In Section 2, we introduce some necessary basic concepts of Bayesian inference, explaining the fMRI data with necessary preprocessing before applying Bayesian methods.  Section 3 describes and compares three Bayesian models for exploring dynamic functions and their case studies.  Section 4 rounds the paper off with a discussion.

## 2. Bayesian Inference

### 2.1. Concept of Bayesian Inference

Bayesian inference is a method of statistical inference in which Bayes' Rule is applied to update the probability estimate for a hypothesis as evidence is acquired, and it is the formal method for combining prior beliefs with observed (quantitative) information to answer the questions that researchers are usually interested in like “what is the probability of getting lung cancer for certain patients who smoke one pack per day?” [37]. It is a natural way to combine multiple experiments' information and it can fit realistic but complicated models.

On the other hand, Bayesian inference often costs more computationally and requires at least one of elicitation of real subjective probability distributions of prior beliefs. The good thing is that sensitivity analysis to show that the choice of prior does not strongly affect inference.

#### 2.1.1. Bayes' Rule [38]

Consider

(1) $$\begin{array}{c} p\left(\;\theta \;|\;y\;\right)=\frac{p\left(y\;|\;\theta \right)p\left(\theta \right)}{\sum_{i}p\left(y\;|\;\theta_{i}\right)p\left(\theta_{i}\right)} \end{array}$$

if *θ* is a discrete random variable with a pmf.

When *θ* is continuous, Bayes' Rule becomes

(2) pθ ∣ ypθ,ypy=py ∣ θpθpy=py ∣ θpθ∫θ py ∣ θpθdθ.

Bayes' Rule is often written as *p*(*θ*∣*y*) ∝ *p*(*θ*)*p*(*y*∣*θ*), when treated as a function of *θ* for a fixed *y*, and *p*(*y*∣*θ*) is the likelihood *L*(*y*∣*θ*). So Bayes' Rule can be thought of as

(3) Posterior∝Prior×Likelihoodpθ ∣ y∝pθpy ∣ θ.

Note that this is expressed in words like “the posterior is proportional to the product of prior and likelihood.”

#### 2.1.2. Basics of the Bayesian Inference [38]

Consider the following:

1. Setting up a probability model.
2. Using the probability theory and the Bayes Rule.

We take binomial model as an example. If the sample size is *n* and the probability of success is *π*, we have *y*∣*π* ~ Bin(*n*, *π*). The likelihood is

(4) $$\begin{array}{c} p\left(\;y\;|\;\pi \;\right)=\left(\begin{array}{c} n\\ y \end{array}\right)\pi^{y}{\left(\;1-\pi \;\right)}^{n-y};\;\pi \in \left[0,1\right]. \end{array}$$

If we want to make inference on *π*, given *y* and *n*, we need a prior distribution *p*(*π*) for *π*. We can choose a uniform distribution:

(5) π~U0,1pπ=10≤π≤10Otherwise.

Then apply Bayes' Rule, and we get

(6) py,π=nyπy1−πn−y,py=∫01nyπy1−πn−ydπ=1n+1,pπ ∣ y=py,πpy=n+1nyπy1−πn−y.

#### 2.1.3. Conjugate Prior [38]

A prior probability distribution is said to be conjugate to the sampling density if the resulting posterior distribution is a member of the same parametric family as the prior. For example, binomial likelihood × beta prior = beta posterior.

### 2.2. Bayesian Analysis Applied to fMRI Data

**Figure 1:**
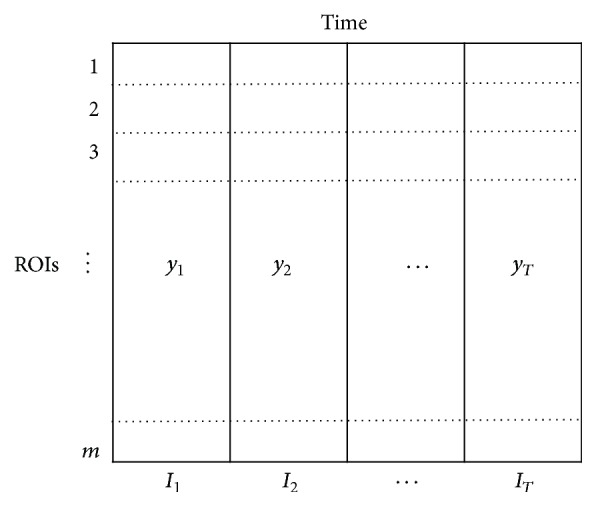
Illustration of data matrix of *Y*, *m* ROIs, and a block indicator vector $\vec{I}$, where *y*_*i*_ is the values of all *m* ROIs at the time point *i*.

An fMRI data series consists of values recorded at a specific voxel of the image at some time point *t*. These series are collected into a *T*-dimensional vector $\vec{y}=(y_{1},y_{2},y_{3},\ldots ,y_{T})$. If the data we collect has *m* regions of interest or *m* neurons based on different experiments, generally our dataset is *m* × *T* matrix *Y*. A simple matrix example is shown in Figure 1.

One of our purposes is to find the dynamic functional connectivity or interaction, in which we need to establish the change point from fMRI data first. In order to apply Bayesian analysis to fMRI data and make inference to the parameters that we are interested in, like finding the change points in the dataset, we need to set up a probability model for the data and find a prior to apply Bayes' Rule.

For example, we want to find the change points in fMRI data and define a block indicator $\vec{I}=(I_{1},I_{2},I_{3},\ldots ,I_{T})$ to indicate the possible locations of change points in the *m* × *T* matrix. Now that the change point indicator is the parameter of interest and we want to make inference to it. We can assume the prior of $\vec{I}$ is Bernoulli (0.5) so that we have $p\left(\vec{I}\right)=\prod_{t=1}^{T}p(I_{t})$.

If $\vec{I}$ is given, we can calculate the likelihood of the fMRI data matrix as $p(Y|\vec{I})$. Sometimes, we need to integrate some nuisance parameter to get this probability.

Thus, the posterior distribution of $p(\vec{I}|Y)$ can be obtained by

(7) $$\begin{array}{c} p\left(\;\vec{I}\;|\;Y\;\right)\propto p\left(\;\vec{I}\;\right)p\left(\;Y\;|\;\vec{I}\;\right). \end{array}$$

After the application of Bayesian analysis theoretically, we design a Markov Chain Monte Carlo (MCMC) scheme to sample the posterior with a random initial block indicator because that is the information we do not know ahead and we want to make inference. So the Bayesian MCMC is applied to the fMRI data. (The MCMC scheme is introduced in details in Section 3.)

Following these procedures, there are several models established and used. In our review, three Bayesian models are elaborated: Bayesian magnitude change point model, Bayesian connectivity change point model, and Bayesian variable partition model for detecting functional interaction and transition patterns, which contains Dynamic Bayesian variable partition model with a two-level MCMC scheme.

In real experiments, before applying the Bayesian methods reviewed here, 358 DICCCOL (Dense Individualized and Common Connectivity-based Cortical Landmarks) [39, 40] ROIs (Regions of Interest) of each subject's brain were first obtained via the publicly available open-source tools in [40] and extracted the fMRI signals. The preprocessing steps of the DTI/R-fMRI (diffusion tensor imaging/resting-state fMRI) images were similar to those used in previous publications [39–43].

## 3. Bayesian-Inference-Based Functional Dynamic Methods

In this section, we first describe two change point detecting models with their one-level MCMC scheme. Next, we introduce another powerful Bayesian model, which is able to infer functional interaction and transition patterns with temporal boundaries identified simultaneously using two-level MCMC scheme. At the end, we summarize these three models and their applications of fMRI data analyses.

### 3.1. Bayesian Magnitude Change Point Model

Lian et al. proposed a Bayesian Magnitude Change Point Model (BMCPM) to detect group-wise consistent magnitude change points on which further pattern recognition of temporal and spatial activations was applied based [35]. A key feature of BMCPM is the capability to consider the group-wise fMRI signals of corresponding cortical landmarks across a population of subjects and optimally determines the change boundaries. In this section, we will elaborate this model in the Bayesian context.

Given a vector $\vec{a}=\left(a_{1},a_{2},\ldots ,a_{t}\right)$ i.i.d. (independent and identically distributed) from a normal distribution, *a* ~ *N*(*μ*, *σ* ^2^), where *t* denotes the dimension of vector $\vec{a}$, *μ* denotes the mean, and *σ* ^2^ denotes the variance. The common way to obtain the posterior distribution of a one-dimensional normal model with unknown mean and variance is to use the conjugate prior Normal-Inverse-Chi-square (*N*-Inv-*χ* ^2^) [44]. In the Bayesian theory, we know that posterior probability ∝ likelihood × prior probability. Since the conjugate prior is used for (*μ*, *σ* ^2^), the posterior distributions are then in the same family as the prior probability distribution. Therefore, assuming a conjugate prior *N*-Inv-*χ* ^2^(*μ*_0_, *σ*_0_^2^/*κ*_0_, *υ*_0_, *σ*_0_^2^) for (*μ*, *σ* ^2^), then the posterior distribution of (*μ*, *σ* ^2^) is the *N*-Inv-*χ* ^2^(*μ*_*t*_, *σ*_*t*_^2^/*κ*_*t*_, *υ*_*t*_, *σ*_*t*_^2^). So the probability of *a*_1_, *a*_2_,…, *a*_*t*_ can be calculated as follows:

(8) pa1,a2,…,at=12πt/2κ0κtΓυt/2Γυ0/2υ0σ02/2υ0/2υtσt2/2υt/2.

Based on (8), given a data matrix $A=\left({\vec{a}}_{1},{\vec{a}}_{2},\ldots ,{\vec{a}}_{m}\right)$, where each ${\vec{a}}_{i}$ is a vector with the data i.i.d. from the normal distribution as described above and ${\vec{a}}_{i}$ is a vector independent from ${\vec{a}}_{j}\;\;(i\neq j)$, the probability of *A* is calculated as

(9) $$\begin{array}{c} p\left(\;A\;\right)=\overset{m}{\underset{i=1}{\prod }}p\left(\;{\vec{a}}_{i}\;\right), \end{array}$$

where $p\left({\vec{a}}_{i}\right)$ is computed according to (8).

**Figure 2:**
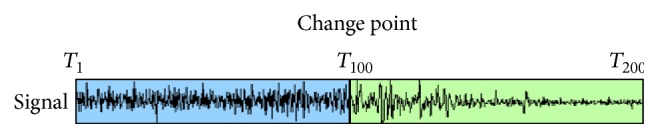
One ROI signal with one magnitude change point at time point *T*_100_.

Magnitude change points are defined as the temporal points dividing ROI data matrix into blocks which exhibit substantial differences in brain states from each other.  Figure 2 demonstrates the basic idea in BMCPM. One temporal change point located at time point *T*_100_ partitions the ROI data matrix into two time blocks with different distributions.

To infer the magnitude temporal change points, we can define a block indicator vector $\vec{I}=\left(I_{1},I_{2},\ldots ,I_{T}\right)$, where *I*_*i*_ = 1 if *i*th observation is the beginning of a block, and otherwise *I*_*i*_ = 0. Therefore, *T* temporal observations were segmented into total ∑_*i*=1_^*T*^ *I*_*i*_ blocks, because *I*_1_ = 1 is always considered as a change point. *Y*_*i*_ is denoted as the observation data of *m* ROIs inside the *i*th. For the fMRI data *Y* defined in Section 2.2, the likelihood of a block indicator vector can be written as follows:

(10) $$\begin{array}{c} p\left(\;Y\;|\;\vec{I}\;\right)=\overset{\sum I_{j}}{\underset{i=1}{\prod }}p\left(\;Y_{i}\;\right), \end{array}$$

where *p*(*Y*_*i*_) can be calculated according to (9). Therefore, the posterior distribution of $\vec{I}$ can be obtained as

(11) $$\begin{array}{c} p\left(\;\vec{I}\;|\;Y\;\right)\propto p\left(\;\vec{I}\;\right)p\left(\;Y\;|\;\vec{I}\;\right). \end{array}$$

We let $p\left(\vec{I}\right)\propto \theta^{\sum I_{j}}{\left(1-\theta \right)}^{\left(T-\sum I_{j}\right)}$ (Bernoulli distribution with parameter *θ*) which may also be modified to reflect our prior knowledge of the estimated number of temporal blocks.

The BMCPM has been successfully applied to the operational span (OSPAN) data, a working memory task-based fMRI dataset with 10 participants, which were acquired on a 3T GE Signa scanner. Totally 81 ROIs were shown with posterior probability larger than 0. Clustering analysis on these 81 ROIs found that some clusters are highly related to specific brain functions. For example, one of the clusters only locates at areas of V1, V2, and motor cortex with well response to low level task-design, while another cluster locates on the frontal or parietal lobes with respect to information processing and responses. More results can be found in [35].

### 3.2. Bayesian Functional Connectivity Change Point Model

In order to analyze the joint probabilities among the nodes of brain networks between different time periods, a Bayesian Connectivity Change Point Model (BCCPM) [34] was proposed to determine the temporal boundary where there is an abrupt change of multivariate functional interactions in the brain networks. Different from BMCPM, which considers ROIs independent of each other, BCCPM infers the boundaries of temporal blocks via a unified Bayesian framework by analyzing the dynamics of multivariate functional interactions.

Given a vector {*b*_1_, *b*_2_,…, *b*_*t*_} i.i.d. from *m*-dimensional multivariate normal distribution $b_{i}\sim N\left(\vec{\mu },\Sigma \right)\;\;i=1,2,\ldots ,t$, where *t* denotes the number of vectors, *m* denotes the dimension of vector *b*_*i*_, $\vec{\mu }$ denotes the *m*-dimensional mean vector, and Σ denotes *m* × *m* the covariance matrix. The common way to obtain the posterior distribution of a multidimensional normal model with unknown mean and covariance matrix is to use the conjugate prior Normal-Inverse-Wishart (*N*-Inv-Wishart) [44]. Therefore, assuming a conjugate prior *N*-Inv-Wishart(*μ*_0_, Λ_0_/*κ*_0_, *υ*_0_, Λ_0_) for $\left(\vec{\mu },\Sigma \right)$, the posterior distribution of $\left(\vec{\mu },\Sigma \right)$ is *N*-Inv-Wishart(*μ*_*t*_, Λ_*t*_/*κ*_*t*_, *υ*_*t*_, Λ_*t*_).

Since we are interested in the posterior distribution of the configuration, the joint probability of *b*_1_, *b*_2_,…, *b*_*t*_ is calculated as follows:

(12) pb1,b2,…,bt=12πmt/2κ0κTm/2Γmνt/2Γmν0/2det⁡Λ0ν0/2det⁡Λtνt/22mt/2,

where Γ_*m*_ is the multivariate gamma function.

**Figure 3:**
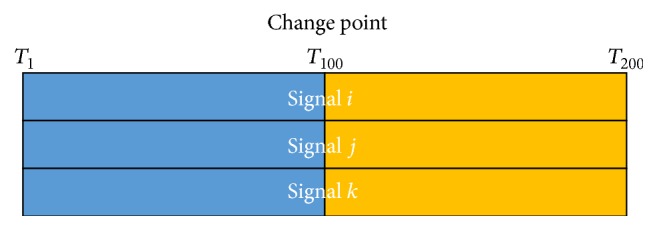
Three ROI signals with one connectivity change point at time point *T*_100_ where the multivariate normal distribution inside the block *T*_1_–*T*_100_ of color blue is different from the distribution of signal in the rest block of color orange.

BCCPM are interested in the connectivity change points, which define the temporal segments where there are underlying differences in the joint probabilities (defining functional interactions) among *m* ROIs between different time-periods.  Figure 3 demonstrates the basic idea in BCCPM. Similar to BMCPM, a block indicator vector is introduced as $\vec{I}=\left(I_{1},I_{2},\ldots ,I_{T}\right)$. The marginal likelihood of the data matrix *Y* = (*y*_1_, *y*_2_,…, *y*_*T*_) can be computed as follows:

(13) $$\begin{array}{c} p\left(\;Y\;|\;\vec{I}\;\right)=\overset{\sum I_{j}}{\underset{i=1}{\prod }}p\left(\;Y_{i}\;\right), \end{array}$$

where *p*(*Y*_*i*_) is calculated according to (12). Please note that one important assumption in the BCCPM is the statistical independence among the temporal segments (blocks). Similar to BMCPM, the posterior distribution of the configuration $p(\vec{I}|Y)$ can be obtained by (11). Note that blocks indicated by $\vec{I}$ are mutually independent to each other across *T*.

BCCPM has been applied to analyze the ADHD data [31], including 25 ADHD-c patients and 49 normal development children as NCs, coming from the Imaging Center for Brain Research, Beijing Normal University. All the ROI time series are partitioned into three blocks with two change points detected by BCCPM. Further analysis based on dictionary learning algorithm identified two pairs of atomic functional interaction patterns. The first pair shared 310 common connections, while the second pair only shared 27 common connections. The matrixes recovered from these two pairs gave 100% discriminations between ADHD patients and NC subjects. More results can be found in [31].

### 3.3. Bayesian Change Point Model Using One-Level MCMC Scheme

Metropolis-Hastings (MH) scheme is widely used for calculating the Bayesian inference. A one-level MH (MCMC) scheme for calculating the posterior distribution under BMCPM or BCCPM is provided as follows with a randomly initialized block indicator vector ${\vec{I}}^{0}$ and user defined iteration number *N*:

(1) Generate a new block indicator vector ${\vec{I}}^{*}$ by randomly switching the value of an element in ${\vec{I}}^{n-1}$. And calculate $p({\vec{I}}^{*}|Y)$ according to (11).
(2) Generate a random number *u* from uniform (0,1) and set
  (14) $$\begin{array}{c} {\vec{I}}^{n}=\left\{\begin{array}{cc} {\vec{I}}^{*} & \text{if }u\leq \min\left[1,\frac{p\left({\vec{I}}^{*}\;|\;Y\right)}{p\left({\vec{I}}^{n-1}\;|\;Y\right)}\right]\\ {\vec{I}}^{n-1} & \text{otherwise.} \end{array}\right. \end{array}$$
(3) Iterate step (1) and step (2) until *n* reaches the given number *N*.
(4) Finally, the posterior probability for each time point (1,2,…, *T*) being a change point is calculated from MCMC samples without burn-in samples.

By default, the control parameters, that is, *μ*_0_, *κ*_0_, *υ*_0_, *σ*_0_^2^, Λ_0_, are fixed as constants. To determine the number of iterations to ensure Markov chains converged with contain configurations, the trace plot of posterior probability and Gelman and Rubin scale reduction factor [45] can be employed.

### 3.4. Dynamic Bayesian Variable Partition Model

Recently, there are several studies which utilized the sliding time window based framework to model multivariate functional connectivity interaction, as well as other works that used graphical modeling methods to detect temporal brain dynamics as mentioned in Section 1. However, it is much needed for an integrated framework to infer the representative signature patterns of the multivariate functional interactions and to simultaneously characterize the temporal transitions of these signature patterns. For this purpose, Zhang et al. [36] proposed a Dynamic Bayesian Variable Partition Model (DBVPM) to simultaneously infer global functional interactions within brain networks and their temporal transition boundaries. To capture all the conditional independence global structure, two dependence structures, that is, chain- and *V*-dependence structures, are designed, which will be first elaborated in the following paragraphs. The DBVPM is trying to simultaneously infer the temporal change points and corresponding global structures inside these temporal blocks.

#### 3.4.1. Chain-Dependence Model

A group of variables *C*_*G*_ follows a chain-dependence model if the index set *G* can be partitioned into three subsets *U*, *V*, and *W* such that *C*_*U*_ and *C*_*W*_ are independent given *C*_*V*_, such as *C*_*U*_ → *C*_*V*_ → *C*_*W*_. The joint distribution of a chain-dependence model is

(15) pCGpCUpCV ∣ CUpCW ∣ CV=FCV,CUFCWCVFCV,

where *F*(*C*_*V*_, *C*_*U*_,…) is the joint probability of (*C*_*V*_, *C*_*U*_,…).

#### 3.4.2. *V*-Dependence Model

A group of variables *C*_*G*_ follows *V*-dependence model if the index set *G* can be partitioned into three subsets *U*, *V*, and *W* such that *C*_*U*_ and *C*_*W*_ are mutually independent, that is *C*_*U*_ → *C*_*V*_ ← *C*_*W*_. The joint distribution of a chain-dependence model is

(16) pCGpCUpCWpCV ∣ CU,CW=FCUFCWFCU,CV,CWFCU,CW,

where *F*(*C*_*V*_,*C*_*U*_,…) is the joint probability of (*C*_*V*_,*C*_*U*_,…). The calculation of this joint probability will be discussed in next section.

#### 3.4.3. Dynamic Bayesian Variable Partition Model

**Figure 4:**
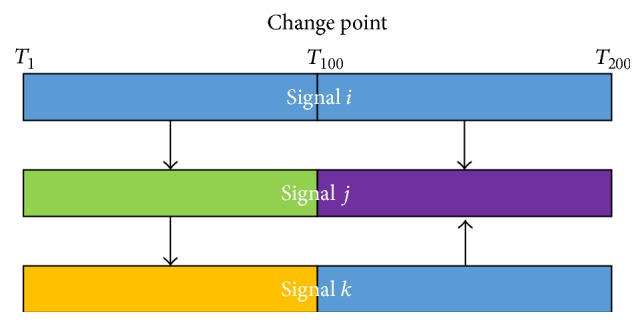
Three ROI signals with one temporal change point at time point *T*_100_ where a chain dependence structure is in the left block and *V* dependence structure is in the right block.

DBVPM uses the similar Bayesian inference as in BCCPM except that the numbers of dimension in the multivariate normal distribution are not fixed across temporal order: specifically, given *c*_1_, *c*_2_,…, *c*_*t*_ i.i.d. observations from the *r*-dimensional multivariate normal distribution, $c_{i}\sim N\left(\vec{\mu },\Sigma \right)\;\;i=1,2,\ldots ,t$. To calculate the marginal distribution of the data *c*_1_, *c*_2_,…, *c*_*t*_, we only need to change the dimension from *m* to *r* in (12), which is the same calculation for the joint probability in (15) and (16), and *r* is determined by the joint of variables.  Figure 4 demonstrates the basic idea in DBVPM.

In DBVPM, to infer the dependence structure (chain or *V* structure) among *m* ROIs, another indicator vector $\vec{\Upsilon }=\left(\Upsilon_{1}{,\Upsilon }_{2},\ldots ,\Upsilon_{m}\right)$ is used to denote the grouping of the index *G* of ROIs to subgroups *U*, *V*, and *W*, where *Υ*_*i*_ = *j* means *i*th ROI is grouped in subgroup *j* (*j* = 0 means *U*, *j* = 1 means *V*, and *j* = 2 means *W*), and a binary indicator *S* is used to denote the dependence structures, that is, chain- or *V*-dependence structure. The posterior distribution for observations in *i*th temporal block can be calculated as

(17) $$\begin{array}{c} p\left(\;\vec{\Upsilon },S\;|\;Y_{i}\;\right)\propto p\left(\;Y_{i}\;|\;\vec{\Upsilon },S\;\right)p\left(\;\vec{\Upsilon }\;\right)p\left(\;S\;\right), \end{array}$$

where *Y*_*i*_ is the same definition in previous sections and *p*(*Y*_*i*_∣*Υ*, *S*) is calculated as (15) when *S* = 0 and (16) when *S* = 1.

To incorporate the multivariate functional interactions and temporal dynamics in DBVPM, the same block indicator $\vec{I}=\left(I_{1},I_{2},\ldots ,I_{T}\right)$ is used. Let $\vec{S}=\left(S_{1},S_{2},\ldots ,S_{\sum I_{i}}\right)$ be the structure indictor vector, and let $\vec{\Upsilon }=\left({\vec{\Upsilon }}_{1},{\vec{\Upsilon }}_{2},\ldots ,{\vec{\Upsilon }}_{\sum I_{i}}\right)$, where ${\vec{\Upsilon }}_{i}=\left(\Upsilon_{1}{,\Upsilon }_{2},\ldots ,\Upsilon_{m}\right)$ with *Υ*_*j*_ = 0,1, 2, be the partition indicator vector in the *i*th block. The posterior distribution of the data matrix *Y* = (*y*_1_, *y*_2_,…, *y*_*T*_) can be represented as follows:

(18) $$\begin{array}{c} p\left(\;\vec{I},\vec{\Upsilon },\vec{S}\;|\;Y\;\right)\propto p\left(\;Y\;|\;\vec{I},\vec{\Upsilon },\vec{S}\;\right)p\left(\;\vec{I},\vec{\Upsilon },\vec{S}\;\right), \end{array}$$

where $p(Y|\vec{I},\vec{\Upsilon },\vec{S})=\prod p(Y_{i}|{\vec{\Upsilon }}_{i},S_{i})$ and $p\left(\vec{I},\vec{\Upsilon },\vec{S}\right)=p\left(\vec{I}\right)\prod p({\vec{\Upsilon }}_{i},S_{i}|I_{i})$. By default, the uniform prior can be used for $p\left(\vec{I}\right)$ and $p({\vec{\Upsilon }}_{i},S_{i}|I_{i})$.

DBVPM has been applied to analyze the posttraumatic stress disorder (PTSD) data with 45 patients and 53 healthy controls coming from the Second Xiangya Hospital and the Central South of University on a 3T MRI (magnetic resonance imaging) scanner. From the difference between the manually labeled change points and the DBVPM-derived time for 98 subjects, it showed that most change points only have 3.5 time-point distance. Further analysis found some substantial state distribution differences based on the detected functional interactions between PTSD patients and healthy controls, which may need additional structural or neural basis investigation in the future. More results can be found in [36].

### 3.5. DBVPM Using Two-Level MCMC Scheme

A two-level MH (MCMC) scheme can be applied to sample from the posterior distribution of the block boundaries and dependency structures within each block: Given the block boundaries, the lower level MCMC samples from the posterior distribution of dependency structures within a block and the higher level MCMC samples from the posterior distribution of block boundaries. Specifically, the lower level MCMC involves alternating between the chain and *V* structures and changing the group labels of each variable of ROI. The likelihood can be calculated using (15), (16), and (18). The higher level MCMC involves segmenting one block into two, merging two neighboring blocks, and shifting a block boundary. In each higher level step, every block runs through a lower level MCMC. A dependency structure is sampled for each block as the dependency structure for that block in the higher level proposal. Then the log likelihood of the proposal can be calculated by summing up the log likelihood of each block. More details can be found in [36].

### 3.6. Bayesian Model Comparison

We conducted a systematical comparison of the above three Bayesian inference models and summarized it in Table 1. The major differences of these models come from their assumptions. In BMCPM, no explicit connection is assumed between ROIs, so the one-dimensional normal model is employed to capture this condition. In BMCPM, all the ROIs are linked together to follow a multi-dimensional normal distribution. In DBVPM, it tries to capture more complex connections between ROIs, so the chain- and *V*-dependence structures are used. As mentioned in Section 1, Bayesian inference methods have been applied to solve three issues in the analysis of fMRI data: detecting magnitude change points; detecting functional connectivities change points; and identifying functional interaction patterns. Since DBVPM uses the chain- and *V*-dependence structures to capture global functional interactions, it owns the power to identify not only all possible change points but also functional interaction patterns, comparing to BMCPM and BCCPM. However, an explicit drawback of DBVPM is the convergence speed that it takes a tremendous time (several weeks on an average power server) to converge when dealing with more than 50 ROIs. This is the reason that selected ROIs from certain brain networks were used in the experiments of real data. We did a simulation test to measure the convergence speed and robust of these three methods. We generated three datasets with 200 time points fixed and the number of ROIs set to 50, 300, and 1000, respectively. The data are generated from the normal distributions, and one change point is embedded at time point 101. Each dataset has five replicas. The iterations are set to 2000 for one-level MCMC and 2000 for both levels for two-level MCMC as suggested by the original studies. We used the machine with the Linux OS, Intel Xeon E5-2620, and 64 GB memory. The average running times (wall clock time) are shown at the end of Table 1. Note that NA means that the method did not finish the processing in three days. Three indicator vectors were used in DBVPM that cause one-level MCMC scheme not suitable for DBVPM. Although BMCPM is only capable of finding magnitude change points, its execution is very fast. Typically, for 358 ROIs, it only needs no more than 2000 iterations and a couple of hours to converge, so it is efficient for handling large fMRI time series data. For BCCPM of multivariate normal distribution assumption, it is good at inference of the boundaries of temporal blocks based on dynamics of multivariate functional interactions. It also converges fast when analyzing data with 358 ROIs. Therefore, if there are some better approaches, like dictionary learning, to further infer functional interactions between ROIs, BCCPM should be the best choice as the first step to establish temporal change points for functional interactions and dynamics.

## 4. Summary and Discussion

In this review paper, we presented and summarized three important applications of the Bayesian inference paradigm to fMRI data. By assuming that normal distribution for each ROI inside temporal block and ROIs are independent of each other, the BMCPM calculates the posterior probability of the temporal block indicator vectors using the conjugate prior of *N*-Inv-*χ* ^2^. The essential feature of BMCPM is the capability of considering the group-wise ROIs in the fMRI signals across a population of subjects and optimally determining the change boundaries. BMCPM converges very fast, and it is able to analyze more than 1000 ROIs, but it only detects magnitude change points. Different from BMCPM considering ROIs independent of each other, the BCCPM assumes that all *m* ROIs are an integrated variable that follows an *m*-dimensional multivariate normal distribution, and thus it can detect function connectivity change points. BCCPM, like BMCPM, only needs one-level MCMC scheme to sample the posterior distribution and converges relatively fast when there are no more than 1000 ROIs. To derive the representative signature patterns of the multivariate functional interactions within temporal blocks, DBVPM was proposed to incorporate two structures, chain- and *V*-dependence structures. Comparing to BMCPM and BCCPM, DBVPM is able to identify the functional interaction patterns in addition to the boundaries of temporal blocks. The cost of DBVPM is that it spends unacceptable time to analyze fMRI data with more than 50 ROIs. The performance of all three Bayesian models were illustrated on three datasets, including task-based OSPAN dataset, attention-deficit/hyperactivity disorder dataset, and posttraumatic stress disorder dataset, which confirmed the superiority of Bayesian inference methods for functional dynamic analysis on fMRI data.

There are still some challenging issues for using Bayesian inference to analyze functional dynamics of fMRI data. For example, from the evaluation of BCCPM on simulation for local dynamics, it showed that BCCPM is not sensitive in detecting small local changes of structure. Also, although global structure inside the temporal blocks can be inferred from DBVPM, the detailed interaction patterns and network structure between ROIs are unknown. Functional connectivity network is also one of the critical applications by evaluating regional interactions using fMRI. One of the possible extensions of BCCPM and DBVPM is to infer the detailed functional connectivity network by estimating the covariance matrix Σ, which could be a valuable future direction for the Bayesian models. As we know, most of the task-based fMRI experiments fall into two categories: (1) block design and (2) event-related design. Currently, the most Bayesian methods are specified and verified for tackling problems based on the data from block design experiments. Comparing to block design, MR (magnetic resonance) signal of efMRI (event-related fMRI) is smaller, and statistical power is relatively lower due to the complexity and the lower SNR (Signal-to-Noise Ratio). The Bayesian model could be a possible tool to analyze event-related design data as well because no explicit assumption is made about what caused the difference in brain connectivity among ROIs. The difference can be originated from either tasks or events. In the future, systematical analyses on simulated and real data are needed to verify the effectiveness and accuracy of Bayesian models for event-based fMRI data, which is an open question and one of the future extensions for Bayesian-inference-based approach.

## Figures and Tables

**Table 1:** Summary of BMCPM, BCCPM, and DBVPM.

	BMCPM	BCCPM	DBVPM
MCMC scheme	One-level	One-level	Two-level
Ability to infer magnitude change points	Yes	Yes	Yes
Ability to infer functional connectivity change points	No	Yes	Yes
Ability to infer functional interaction patterns	No	No	Yes
Running time			
50 ROIs, 200 time points	2 seconds	49 seconds	948 minutes
300 ROIs, 200 time points	11 seconds	41 minutes	NA
1000 ROIs, 200 time points	36 seconds	483 minutes	NA
